# Evaluation of diffusion‐weighted MRI and geometric distortion on a 0.35T MR‐LINAC at multiple gantry angles

**DOI:** 10.1002/acm2.13135

**Published:** 2021-01-15

**Authors:** Benjamin Lewis, Anamaria Guta, Stacie Mackey, H. Michael Gach, Sasa Mutic, Olga Green, Taeho Kim

**Affiliations:** ^1^ Departments of Radiation Oncology Washington University School of Medicine in St. Louis St. Louis MO USA; ^2^ Department of Radiation Oncology Barnes Jewish Hospital St. Louis MO USA; ^3^ Departments of Radiology Washington University School of Medicine in St. Louis St. Louis MO USA; ^4^ Department of Biomedical Engineering Washington University in St. Louis St. Louis MO USA

**Keywords:** DWI, MRgRT, MR‐LINAC

## Abstract

Diffusion‐weighted imaging (DWI) provides a valuable diagnostic tool for tumor evaluation. Yet, it is difficult to acquire daily MRI data sets in the traditional radiotherapy clinical setting due to patient burden and limited resources. However, integrated MRI radiotherapy treatment systems facilitate daily functional MRI acquisitions like DWI during treatment exams. Before ADC values from MR‐RT systems can be used clinically their reproducibility and accuracy must be quantified. This study used a NIST traceable DWI phantom to verify ADC values acquired on a 0.35 T MR‐LINAC system at multiple gantry angles. A diffusion‐weighted echo planar imaging sequence was used for all image acquisitions, with b‐values of 0, 500, 900, 2000 s/mm^2^ for the 1.5 T and 3.0 T systems and 0, 200, 500, 800 s/mm^2^ for the 0.35 T system. Images were acquired at multiple gantry angles on the MR‐LINAC system from 0° to 330° in 30° increments to assess the impact of gantry angle on geometric distortion and ADC values. CT images, and three fiducial markers were used as ground truth for geometric distortion measurements. The distance between fiducial markers increased by as much as 7.2 mm on the MR‐LINAC at gantry angle 60°. ADC values of deionized water vials from the 1.5 T and 3.0 T systems were 8.30 × 10^‐6^ mm^2^/s and −0.85 × 10^‐6^ mm^2^/s off, respectively, from the expected value of 1127 × 10^‐6^ mm^2^/s. The MR‐LINAC system provided an ADC value of the pure water vials that was −116.63 × 10^‐6^ mm^2^/s off from the expected value of 1127 × 10^‐6^ mm^2^/s. The MR‐LINAC also showed a variation in ADC across all gantry angles of 33.72 × 10^‐6^ mm^2^/s and 20.41 × 10^‐6^ mm^2^/s for the vials with expected values of 1127 × 10^‐6^ mm^2^/s and 248 × 10^‐6^ mm^2^/s, respectively. This study showed that variation of the ADC values and geometric information on the 0.35 T MR‐LINAC system was dependent on the gantry angle at acquisition.

## 1 INTRODUCTION

As MR‐guided radiotherapy (MRgRT) continues to develop, additional tools are being adapted from diagnostic MRI systems to combined MRI‐Radiotherapy treatment (MR‐RT) systems to provide more information to clinical teams. Currently, combined MR‐RT systems provide imaging in the treatment position with superior soft tissue contrast, compared to x‐ray imaging, real‐time tumor tracking with CINE imaging, beam gating, and the ability to perform daily adaptive radiotherapy (ART).^1^, ^2^, ^3^, ^4^ Diffusion‐weighted imaging (DWI) is an important imaging biomarker for tumor identification and assessment of response to radiotherapy that can indicate changes in tumor function before tumor size or morphology changes appear on traditional imaging methods.^5^, ^6^, ^7^, ^8^, ^9^ Changes in the apparent diffusion coefficient (ADC) of tumors were correlated with local tumor control and radiotherapy treatment outcomes.^10^, ^11^, ^12^, ^13^ Therefore, DWI is an excellent tool for ART, allowing a patient’s treatment plan to be adapted based on improved visualization of the tumor with DWI, and quantitative information from the calculated ADC.

However, performing imaging studies to monitor treatment response requires significant dedication of imaging resources and increases the patient burden. Very few studies have been performed to monitor changes in ADC values over time and often are performed at different time points during treatment.^14^, ^15^, ^16^ MR‐RT systems allow daily imaging during treatment, including DWI, without significant changes to the standard treatment protocol. Direct application of ADC values from dedicated MRI systems to MR‐RT systems may be difficult due to substantial differences in the design of the MRI components compared to conventional diagnostic systems. Therefore, the difference between diagnostic MRI and MR‐RT system ADC values must be assessed before DWI can be used as a functional imaging tool for treatment response monitoring or ART. The feasibility of DWI acquisition for a 0.35 T MR‐^60^Co system and for a 1.5 T MR‐LINAC system was shown in prior studies.^17^, ^18^ An added challenge presented by the MR‐LINAC system is the motion of the LINAC gantry around the MRI. Although there is significant magnetic and RF shielding between the MRI and LINAC subsystems, changes in the gantry angle cause changes in the imaging isocenter and spatial integrity.^19^ These changes could adversely impact the accuracy of ADC values and reduce their utility for ART or disease monitoring.

In this study, we perform a phantom‐based accuracy and reproducibility study of ADC values calculated from DWI MRIs acquired on clinical 1.5 T and 3.0 T MRI systems, and on a 0.35 T MR‐LINAC MR‐RT system with multiple gantry angles.

## 2 METHODS

DWI MRIs and ADC values were quantified using a phantom‐based approach. A diagnostic 3.0 T Siemens Vida scanner (Erlangen, Germany) and a radiation oncology MR‐simulator 1.5 T Philips wide bore Ingenia scanner (Amsterdam, Netherlands) were used for comparison and to confirm ADC value calculation accuracy. The same phantom was then imaged on the ViewRay MRIdian 0.35 T MR‐LINAC system (Mountain View, CA, USA).^20^, ^21^

### 2.1 DWI phantom preparation

The CaliberMRI Diffusion Standard Model 128 (Caliber MRI, Boulder, CO, USA) was used for all image acquisitions. The diffusion phantom body is a spherical shell with 13 vials containing aqueous solutions of the polymer polyvinylpyrrolidone (PVP) in concentrations of 0% (vials 1–3), 10% (vials 4–5), 20% (vials 6–7), 30% (vials 8–9), 40% (vials 10–11), and 50% (vials 12–13) which were labeled as concentrations 1 through 6. The ADC values for each solution at 0°C were provided with the phantom and are NIST traceable.^22^

The day before imaging occurred the phantom was filled with distilled water and ice then placed into a refrigerator overnight. Immediately prior to imaging additional ice was added to the phantom and it was placed into a cooler with ice for transport to ensure the phantom remained at or near 0°C during imaging. The water temperature was acquired with a NIST traceable long stem digital thermometer (Thomas Scientific, precision = 0.01°C, accuracy = ±0.005°C) through the top fill port. Temperature was taken before and after all image acquisitions with the maximum temperature after image acquisition being less than 1°C for all acquisitions.

### 2.2 Image acquisition

As the vendor of the phantom recommended, images were acquired with diffusion‐weighted echo‐planar‐imaging (DW‐EPI). The highest b‐values were reduced on the 0.35 T system due to gradient limitations. Three independent imaging sessions were performed on each scanner. The diffusion phantom was also imaged on a Philips wide bore CT scanner to provide a ground truth geometric dataset using the following parameters: coronal orientation, field of view (FOV) = 373 × 373 × 221.5 mm^3^, matrix size = 512 × 512 × 443, slice spacing = −0.5 mm.

#### 2.2.1 Siemens and Philips MRI systems

The diffusion phantom was imaged at 1.5 and 3 T to verify the ADC calculation methodology and ensure that the calculated values matched the values provided by the manufacturer of the phantom. Three independent scans were acquired on each MRI system. Diffusion images were acquired with four b‐values (0, 500, 900, and 2000 s/mm^2^). Images acquired on the 3.0 T system used a three‐scan trace echoplanar spin echo (EPSE) diffusion‐weighted sequence, with the following parameters: orientation = coronal, FOV = 243 × 243 mm^2^, TR = 10,000 ms, TE = 80 ms, flip angle = 90°, matrix size = 192 × 192, voxel size = 1.1 × 1.1 mm^2^, number of slices = 25, slice thickness = 5 mm, number of averages = 1, readout bandwidth = 1042 Hz/Pixel, echo train length (ETL) = 71, GRAPPA parallel acquisition, in‐plane parallel reduction factor = 2, partial Fourier number = 6/8, partial Fourier direction = phase, and a 20‐channel head and neck coil. The images acquired on the 1.5 T system used a diffusion‐weighted EPSE sequence with the following parameters: orientation = coronal, FOV = 220 × 220 mm^2^, TR = 10,000 ms, TE = 107 ms, flip angle = 90°, acquisition matrix size = 128 × 126 ( acquisition voxel size = 1.72 × 1.72 mm^2^), reconstruction matrix size = 256 × 256 (reconstruction voxel size = 0.898 × 0.898 mm^2^), number of slices = 25, slice thickness = 4 mm, number of averages = 1, readout bandwidth = 1645 Hz/Pixel, echo train length (ETL) = 63, SENSE parallel acquisition, in‐plane parallel reduction factor = 2, partial Fourier number = 6/8, partial Fourier direction = phase, and a head and neck multicoil.

#### 2.2.2 ViewRay system

The diffusion phantom was imaged on the ViewRay MRIdian 0.35 T MR‐LINAC system in MRI QA mode at 12 gantry angles spaced every 30° from 0° to 330°. Three independent imaging sessions were acquired. The phantom was placed in the daily QA phantom cradle with the anterior and posterior torso receiver RF coils in place. Diffusion images were acquired with four b‐values (0, 200, 500, and 800 s/mm^2^). Images were acquired with an EPI diffusion weighted sequence with the following parameters: orientation = coronal, field of view (FOV) = 300 × 300 mm^2^, TR = 5500 ms, TE = 124 ms, flip angle = 90°, matrix size = 128 × 128, voxel size = 2.34 × 2.34 mm^2^, number of slices = 3, slice thickness = 5 mm, number of averages = 4, readout bandwidth = 1628 Hz/Pixel, echo train length (ETL) = 96, partial Fourier direction = phase, partial Fourier number = 6/8, parallel imaging disabled, and total acquisition time = 94 s. A full prescan shimming sequence was performed prior to imaging at each gantry angle.

### 2.3 Geometric distortion quantification

**Fig. 1:**
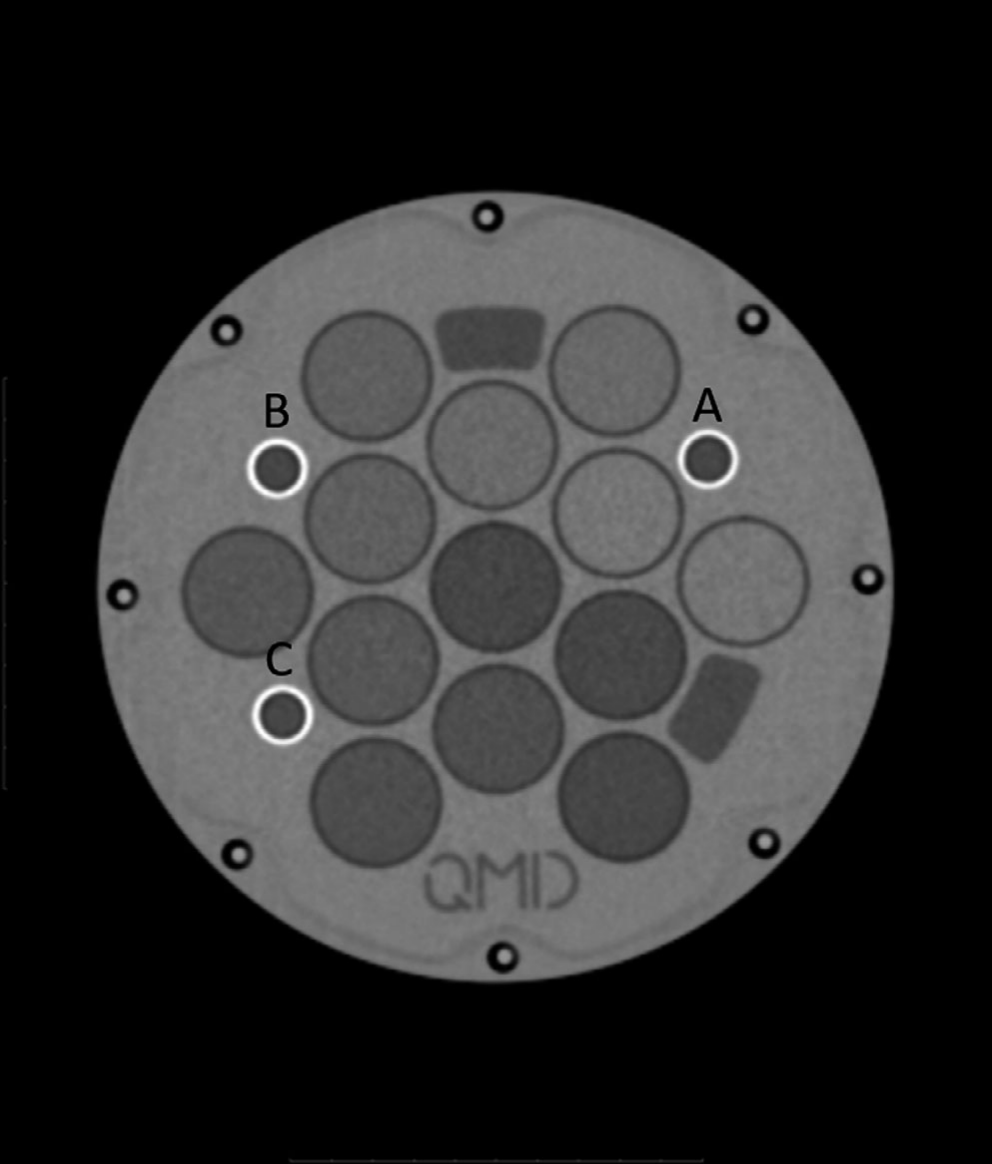
CT image of DWI phantom CT image of the central slice of the DWI phantom with the fiducial markers labeled as a, b, and c.

To assess geometric distortion, the CT images were taken as the ground truth. The DWI phantom includes three fixed fiducial markers, attached to the central plate of the phantom. The fiducial markers were labeled A, B, and C as shown in Fig. 1. The distances from A to B, B to C, and A to C were measured using the Philips multimodality DICOM Viewer software (Philips Medical Systems, Netherlands).

### 2.4 ADC value calculation

After image acquisition, the same image processing was performed on images from all three systems with in‐house scripts using MATLAB 2019b (MathWorks, Natick, MA, USA). The noise floor was removed to reduce the impact of noise on the ADC calculation using the noise subtraction method proposed by Dietrich et al.^23^ using the following equation:

(1) $$S_{\mathrm{nc}}=\sqrt{\left|S_{n}^{2}‐\frac{2}{\pi }N^{2}\right|},$$

where *S_nc_* and *S_n_* are the noise corrected and noisy images, respectively, *N* is the average background noise signal intensity from a consistently positioned ROI. Using purpose built MATLAB scripts, all b‐value images were then registered to the b0 image using affine registration to correct eddy current distortion, and then registered to the corresponding slice from images acquired at the home gantry angle using affine registration to remove geometric distortion caused by gantry position. The home gantry angle served as the fixed image for 2D‐affine geometric transformation to correct for gantry position related distortions because it is the gantry position used for generating the standard shim map at installation of the machine. Then ground truth geometric correction was performed using affine registration of images corrected to the home gantry position to the acquired CT at the corresponding slice location. CT images served as the ground truth geometric image to correct geometric distortion caused by eddy currents and imperfect shimming. ADCs were then calculated for ROIs in the center of each vial using least‐squares fitting of all b‐values to the following equation:

(2) $$S_{\mathrm{nc}}\left(b\right)=S_{0}e^{‐b*ADC},$$

where *S_nc_(b)* is the noise corrected pixel signal intensity, *b* is the image b‐value, and *S_0_* is the signal intensity for b = 0 s/mm^2^ image. ADC values are reported as averages for each concentration. Statistical significance of ADC value variation was evaluated in a two‐step process. First, a one‐way ANOVA test was applied for each concentration level, with a significance level of *P* < 0.05. Concentrations that had a statistically significant variance were then evaluated with a Tukey’s range test to determine which gantry angles had significantly different mean values.

## 3 RESULTS

**Fig. 2:**
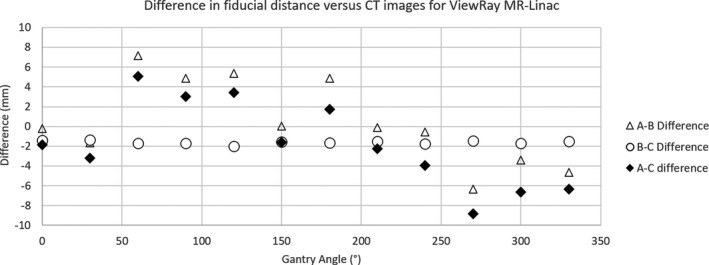
Phantom geometric distortion. The difference in distance between fiducial markers measured on CT versus the ViewRay MR‐LINAC at different gantry angles from 0° to 330° in 30° intervals.

On CT images the distances between the fiducials were 104.5, 60, and 120.4 mm from A‐B, B‐C, and A‐C, respectively. The average differences between fiducial distances measured on CT vs MRI are shown in Table 1. The difference in fiducial distance for different gantry angles measured on the MR‐LINAC system is shown in Fig. 2.

**Table 1:** The difference in distance between fiducials measured on the CT vs the indicated MRI system, averaged over the three independent imaging sessions. For the MR‐LINAC system, images are from the gantry 0° position only.

	A‐B Difference (mm)	B‐C Difference (mm)	A‐C Difference (mm)
Siemens 3.0 T	‐0.8 ± 0.3	‐0.3 ± 0.3	‐1.0 ± 1.0
Philips 1.5 T	‐0.1 ± 0.5	‐0.3 ± 0.4	‐0.2 ± 0.8
ViewRay MR‐LINAC (Gantry 0°)	‐0.2 ± 0.2	‐1.4 ± 0.4	‐1.9 ± 0.2

**Fig. 3:**
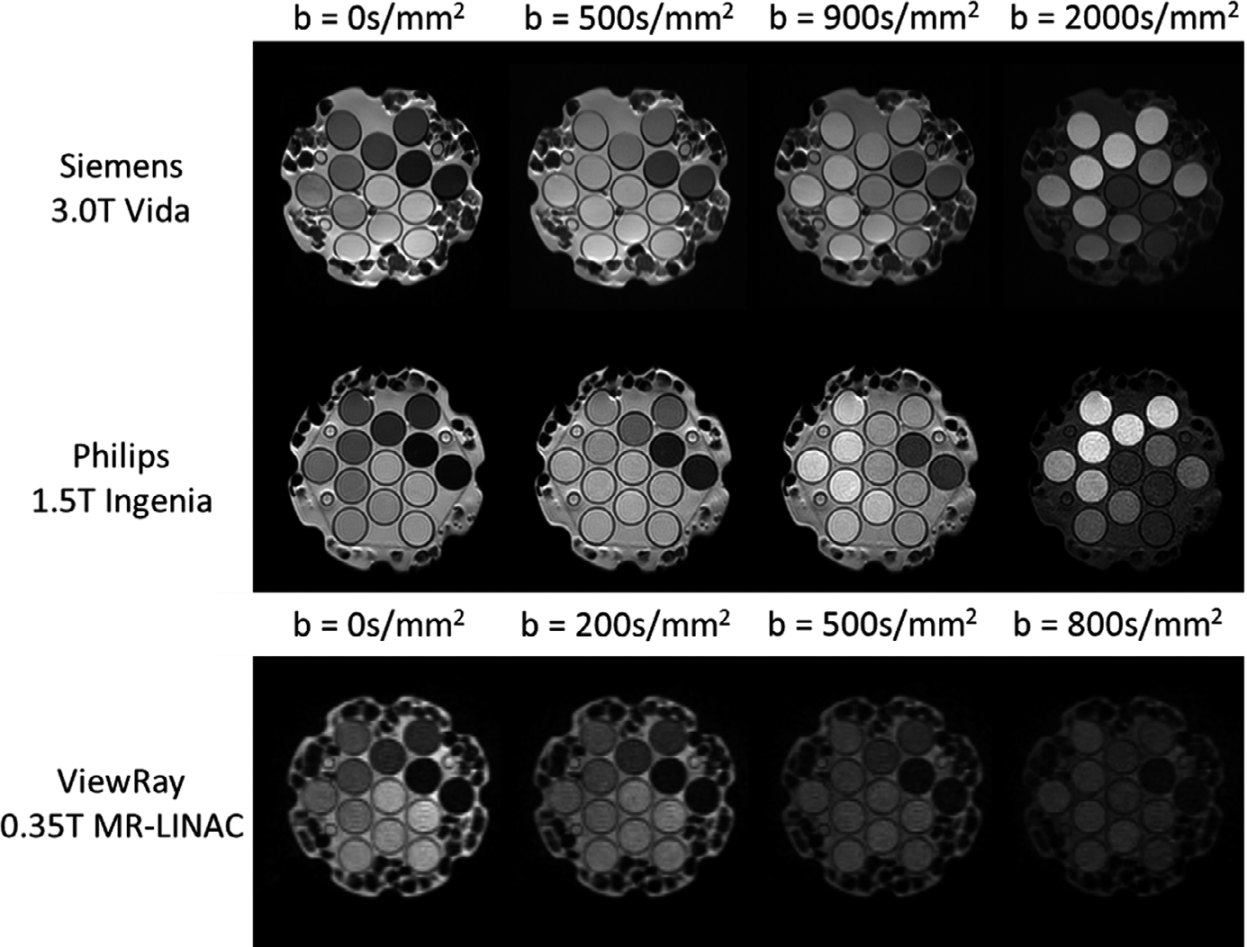
MRI phantom DWI images. DWI images acquired on all three systems. Rows from top to bottom are the 3.0 T Siemens Vida, 1.5 T Philips wide bore Ingenia, and 0.35 T ViewRay MR‐LINAC systems. Columns from left to right are images with b‐values of 0, 500, 900, 2000 s/mm^2^ for the 1.5 T and 3.0 T systems, and b‐values of 0, 200, 500, 800 s/mm^2^ for the 0.35 T system.

The DWI images acquired on each machine are shown in Fig. 3. Calculation of ADC values from images acquired on 1.5 T and 3.0 T systems served as a verification of the ADC calculation methodology and was compared against the NIST traceable ADC values provided by the manufacturer. ADC values from images acquired at 3.0 T differed from the expected values by −0.85 × 10^‐6^ mm^2^/s and 6.12 × 10^‐6^ mm^2^/s for vial concentrations 1 (ref = 1127 × 10^‐6^ mm^2^/s) and 5 (ref = 248 × 10^‐6^ mm^2^/s), respectively. The 1.5 T system provided ADC values with a greater deviation from expected with the difference from expected values being 8.30 × 10^‐6^ mm^2^/s and −21.62 × 10^‐6^ mm^2^/s for vial concentrations 1 and 5, respectively. The calculated ADC values from the 1.5 T and 3.0 T systems are shown in Table 2.

**Table 2:** ADC values from the 1.5 T and 3.0 T systems at 0°C for all six vial concentrations.

	Reference (x10^‐6^ mm^2^/s)	1.5 T			3.0 T		
		ADC (x10^‐6^ mm^2^/s)	Difference from Ref. (x10^‐6^ mm^2^/s)	% Difference from Ref.	ADC (x10^‐6^ mm^2^/s)	Difference from Ref. (x10^‐6^ mm^2^/s)	% Difference from Ref.
Conc. 1	1127 ± 1	1135.30 ± 25.01	8.30	0.1%	1126.15 ± 13.10	‐0.85	‐0.08%
Conc. 2	843 ± 3	839.35 ± 5.23	‐3.65	‐0.4%	869.41 ± 11.33	26.41	3.1%
Conc. 3	607 ± 2	592.08 ± 7.25	‐14.92	‐2.5%	602.86 ± 6.67	‐4.14	‐0.7%
Conc. 4	403 ± 5	381.36 ± 9.95	‐21.64	‐5.4%	392.61 ± 6.87	‐10.39	‐2.6%
Conc. 5	248 ± 6	226.38 ± 9.98	‐21.62	‐8.7%	254.12 ± 16.15	6.12	2.5%
Conc. 6	128 ± 8	117.97 ± 8.45	‐10.03	‐7.8%	122.09 ± 20.66	‐5.91	‐4.62%

**Fig. 4:**
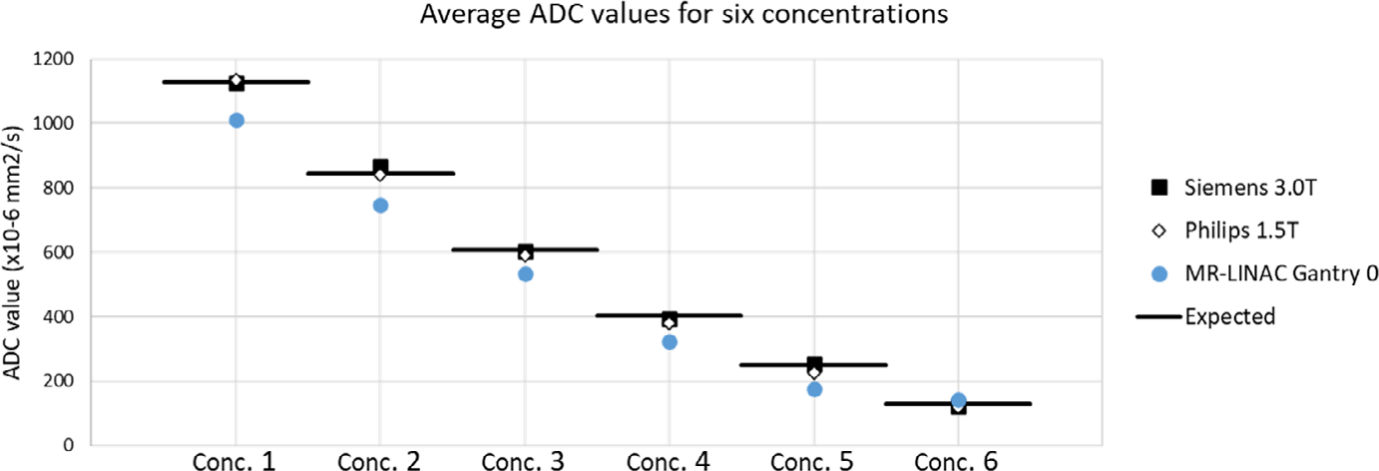
ADC value comparison. ADC values averaged over the three imaging sessions for each MRI system. The black bars indicate the reference ADC values. Conc. indicates the vial concentration designation.

On the 0.35 T ViewRay system, the calculated ADC values were consistently and significantly lower than the expected values and the 1.5 T and 3.0 T systems. For vial concentrations 1 and 5, the MR‐LINAC system at home position, produced ADC values that were −116.63 × 10^‐6^ mm^2^/s and −71.58 × 10^‐6^ mm^2^/s different from the reference values of 1127 × 10^‐6^ mm^2^/s and 248 × 10^‐6^ mm^2^/s, respectively. The ADC values for all three systems are shown in Fig. 4. Values for the MR‐LINAC system are at the 0° gantry position.

**Fig. 5:**
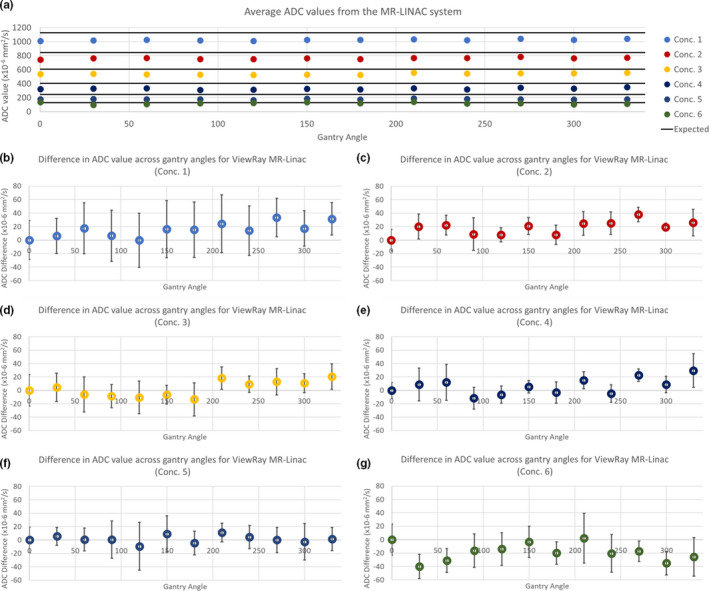
ADC gantry angle dependence. (a) shows the ADC values averaged over three scanning sessions at gantry angles 0° to 330° at 30° increments. The black bars indicate the reference ADC value for each concentration. (b) through (g) show the difference in ADC values relative to the home gantry angle with error bars indicating ±1 standard deviation for all vial concentrations.

Across gantry angles the MR‐LINAC system produced ADC values that varied by 33.72 × 10^‐6^ mm^2^/s for vial concentration 1 with a reference value of 1127 × 10^‐6^ mm^2^/s, and 20.41 × 10^‐6^ mm^2^/s for vial concentration 5, which had a reference value of 248 × 10^‐6^ mm^2^/s. The ADC values over multiple gantry angles are shown in Fig. 5. Table 3 shows the percent difference from reference value for all concentrations and measured gantry angles. A two‐tailed paired t‐test analysis was performed in MATLAB for all concentrations to assess the difference between ADC values acquired at different angles. Vial concentrations 2 and 4 showed a statistically significant variance in ANOVA testing (*P* < 0.05), p‐values for all concentrations are shown in Table 4. Tukey’s range test showed significant differences in the means of vial concentration 2 for gantry angles 0° and 270°, while vial concentration 4 was between gantry angles 90° and 330°. Geometric correction with affine registration and noise floor subtraction improved ADC value agreement with the reference value from an average of −12.0% to −9.0 and −32.9% to −28.3% for vial concentrations 1 and 5, respectively, across all gantry angles. The highest concentration vials (concentration 6, 50% PVP) produced ADC values similar to the reference value. However, the diffusivity of concentration 6 (127 × 10^‐6^ mm^2^/s) was below physiological values and vial signal intensities approached the noise floor for images acquired with b‐values 500 s/mm^2^ and above on the 0.35 T ViewRay system.

**Table 3:** The percent difference from ADC reference value acquired on the 0.35 T MR‐LINAC at 0°C for all six vial concentrations and all gantry angles.

Gantry angle (°)	Percent difference from concentration reference values (%)					
	Conc. 1	Conc. 2	Conc. 3	Conc. 4	Conc. 5	Conc. 6
0	‐10.3%	‐11.8%	‐11.5%	‐20.2%	‐28.9%	8.3%
30	‐9.8%	‐9.3%	‐10.7%	‐18.0%	‐26.6%	‐23.1%
60	‐8.8%	‐9.1%	‐12.5%	‐17.2%	‐28.6%	‐16.2%
90	‐9.8%	‐10.7%	‐12.9%	‐23.1%	‐28.6%	‐4.6%
120	‐10.4%	‐10.8%	‐13.2%	‐21.8%	‐32.6%	‐2.7%
150	‐8.9%	‐9.3%	‐12.5%	‐18.8%	‐25.2%	5.6%
180	‐9.0%	‐10.8%	‐13.7%	‐21.0%	‐30.7%	‐7.3%
210	‐8.2%	‐8.8%	‐8.4%	‐16.5%	‐24.4%	9.9%
240	‐9.1%	‐8.8%	‐10.0%	‐21.4%	‐27.1%	‐7.7%
270	‐7.4%	‐7.2%	‐9.3%	‐14.6%	‐28.8%	‐5.2%
300	‐8.8%	‐9.5%	‐9.7%	‐18.0%	‐30.0%	‐19.1%
330	‐7.6%	‐8.7%	‐8.1%	‐12.8%	‐28.3%	‐11.8%

**Table 4:** The *P*‐ values from a one‐sided ANOVA test of ADC values of each vial concentration across the 12 gantry angles.

	Conc. 1	Conc. 2	Conc. 3	Conc. 4	Conc. 5	Conc. 6
*P*‐value	0.658824	**0.028514**	0.089786	**0.007413**	0.788532	0.121875

Statistically significant values (*P* < 0.05) are displayed in bold and gray cells.

## 4 DISCUSSION

To the best of our knowledge, this is the first evaluation of ADC values at multiple gantry angles on the 0.35 T ViewRay MR‐LINAC system. This work demonstrated that ADC values do change with gantry angle with an EPI sequence following noise floor removal and geometric distortion correction. The ADC values produced by the 0.35 T system in this study were also significantly lower than those produced on 1.5 T and 3.0 T systems. Additionally, geometric distortion assessment using fiducial markers showed a gantry dependent distortion, similar to those reported by *Kim et al*.^19^ for anatomical imaging sequences used during patient treatment on the MR‐LINAC system.

The significant difference in ADC values between high field systems and the 0.35 T system suggests that further characterization and ADC value correction needs to be performed before ADC values acquired on the MR‐LINAC system can be applied as quantitative functional values in the clinical setting. Geometric distortion caused by eddy currents, gantry position, and the EPI sequence were corrected. However, this was not sufficient to produce correct ADC values. After geometric correction, the ADC values were changed by +3% and +4.6% of the reference value for vials 1 and 5, respectively, for gantry at 0°. Low SNR and high noise floor can also impact the accuracy of ADC values. Signal averaging was used for low field acquisition to improve SNR to an acceptable level. However, utilizing multiple signal averages for human imaging may introduce additional errors due to motion during scan acquisition. *Dietrich et al*. also has shown that the benefit of increasing NSA is limited. Increasing NSA will reduce the fluctuation caused by random signal intensity values but cannot reduce the ADC shift introduced by Rician noise inherent to diffusion‐weighted acquisitions. Multiple signal averaging resulted in concentration 1 having a signal intensity eight and four times greater than background for the b = 0 s/mm^2^ and b = 800 s/mm^2^ images, respectively, after postprocessing. Furthermore, the SNR was greater than 40 for concentration 1 in the b = 0 s/mm^2^ images. Previous studies have shown that SNR values greater than 20 are sufficient to accurately measure ADC values with DWI sequences.^24^, ^25^ Concentration 6 was the only concentration that had a signal intensity equivalent to the noise floor at high b‐values on the 0.35 T MR‐LINAC. The low signal intensity of concentration 6 is likely the cause of the good agreement of ADC values to the reference value, as seen in Table 3, with residual noise generating an artificial ADC value. Calculated ADC values did not match the ADCL traceable reference values on the 0.35 T MR‐RT system with the vendor specified measurement temperature and phantom setup, but images had sufficient SNR for accurate ADC calculation and geometric corrections applied. Another source of ADC value error may be from the specified b‐values not being achieved during diffusion weighted EPI acquisition. This issue may be further exacerbated on the MR‐LINAC when the gantry angle is varied, which was shown to cause changes in phantom geometry and ADC values in this study. Motion of the gantry alters the position of ferromagnetic devices around the MR system, causing signal intensity changes even though full prescan shimming was performed for each new gantry \angle, indicating achieved b‐values may be altered by the gantry position. Further work is required to investigate if diffusion gradient performance varies across gantry angles or if the desired b‐values are being achieved, it is presented here as an alternate explanation for ADC discrepancies that were not resolved through geometric and noise corrections.

Diffusion images and ADC values can still be utilized as a qualitative assessment of tumor change over time during treatment. For example, tumors with high cellularity will show high signal intensity on high b‐value images. If the signal intensity falls with treatment that could indicate a reduction in cellularity and damage to the tumor cells. Diffusion‐weighted images and ADC maps can also be early indicators of fibrosis and edema in tissue. For better geometric accuracy, DWI images should be acquired at gantry angle 0°, however, this location may by system dependent and should be assessed for individual machines. This is different than the home gantry angle designated during acceptance and commissioning determined by anatomical imaging isocenter shift measurements for this MR‐LINAC system.

Further study is required to generate quantitative ADC maps with an EPI sequence on the ViewRay 0.35 T MR‐LINAC system. This may include changes to pulse sequence TE and receiver bandwidth to improve signal to noise ratio (SNR) and utilizing a different set of acquisition b‐values to prevent signal washout for low ADC volumes. Image acquisition on the MR‐LINAC system was performed in MRI QA mode because DWI had not been activated in clinical mode by ViewRay. Performing acquisitions in MRI QA mode also allowed for control over all image parameters and a standardized parameter set.

Additional DWI methods are being investigated by *Gao et al*., using a diffusion‐prepared turbo spin echo (DP‐TSE) readout sequence in place of the DW‐EPI sequence. They showed promising results in producing distortion free diffusion‐weighted images with accurate ADC values.^17^ Our study only utilized the DW‐EPI sequence because it is a standard protocol that was recommended by the diffusion phantom vendor, and allowed for direct comparison between the MR‐RT system and diagnostic scanners. Another source of error may be hardware limitations resulting in inconsistent gradient field strengths. However, that is outside the scope of this work and would require specialized equipment.

This study had some limitations, including the phantom itself. The phantom required the addition of ice to the water in the internal basin, this resulted in significant susceptibility‐related heterogeneities. The heterogeneities produced large artifacts on 1.5 T and 3.0 T systems. However, they were greatly reduced on the 0.35 T system. The coronal orientation was chosen for image acquisition, instead of the transverse orientation typical for patient imaging, because it prevented air bubbles trapped within the vials from being within the imaging plane. The larger imaging volume required for human imaging may also increase magnetic inhomogeneity and further degrade gradient fields for diffusion imaging.

This work demonstrated the gantry dependent geometric distortion of a DWI phantom, as well as gantry dependence of calculated ADC values with a methodology verified with 1.5 T and 3.0 T scanners, as well as NIST traceable diffusion values provided by the manufacturer.

## 5 CONCLUSION

ADC values of a NIST traceable phantom were calculated and compared on 3.0 T Siemens, 1.5 T Philips, and 0.35 T ViewRay MR‐LINAC systems. ADC calculations showed good agreement between the vendor provided values and the ≥1.5 T MRI systems, while the low‐field ViewRay system produced ADC values significantly lower than the reference values. Additionally, DWI images acquired at different gantry angles on the MR‐LINAC showed variability in ADC values.
